# Parent Preferences for Acute Respiratory Tract Infection Care

**DOI:** 10.1001/jamanetworkopen.2025.25904

**Published:** 2025-08-08

**Authors:** Janel Hanmer, Sarah K. Burns, Samuel R. Wittman, Tran T. Doan, Tamar Krishnamurti, Kristin N. Ray

**Affiliations:** 1 Department of Medicine, University of Pittsburgh School of Medicine, Pittsburgh, Pennsylvania; 2 Department of Pediatrics, University of Pittsburgh School of Medicine (UPMC), UPMC Children’s Hospital of Pittsburgh, Pittsburgh, Pennsylvania; 3 Department of Health Systems, Management, and Policy, Colorado School of Public Health, Aurora

## Abstract

**Importance:**

Care is commonly sought for pediatric acute respiratory tract infections (ARTIs). The site where families seek ARTI care affects continuity, quality, and costs, but how parents of young children balance tradeoffs between care sites is unknown.

**Objectives:**

To evaluate preferences for attributes of ARTI care sites among parents of young children using a discrete choice experiment (DCE).

**Design, Setting, and Participants:**

This survey study using a DCE was conducted among a nationally representative panel of US parents of children aged 6 months to 5 years between July 31 and August 18, 2023. Participants completed a DCE survey with 7 care attributes (visit modality, visit timeliness, noncare time, out-of-pocket costs, clinician continuity, pediatric expertise, and follow-up availability), with 2 to 5 levels each. The DCE used standard choice-based conjoint procedures. Sawtooth software was used to create a random, balanced overlap experimental design. Data were analyzed between October 2023 and October 2024.

**Main Outcomes and Measures:**

The main outcomes were preferences for attributes and their levels, examined using hierarchical bayesian and latent class analyses and market simulation of care choices under different scenarios.

**Results:**

Of 944 respondents, 63 (6.7%) self-identified as Asian or Pacific Islander, 228 (24.2%) as Hispanic, 120 (12.8%) as Black non-Hispanic, 501 (53.1%) as White non-Hispanic, and 30 (3.3%) as other non-Hispanic; 53% were women; 285 spoke a language other than English at home; and 338 had children who received Medicaid. Respondents’ mean (SD) age was 36.5 (7.4). Overall, respondents gave most weight to visit modality (in person vs telemedicine; importance, 22.7%) and out-of-pocket costs (importance, 23.7%) and least weight to noncare time (importance, 5.8%) and availability of follow-up within 2 days (importance, 6.8%). Latent class analysis identified 4 subgroups: urgency focused (47.4% of respondents), continuity focused (22.7%), cost focused (20.6%), and in-person visit focused (9.3%). The addition of a telehealth visit option with the child’s usual primary care practice available within 30 minutes to options of in-person primary care, urgent care, and emergency department care shifted reduced anticipated care seeking outside of primary care in urgency-focused (−13.3%) and cost-focused (−5.6%) subgroups and reduced forgone care in the cost-focused subgroup (−8.1%).

**Conclusions and Relevance:**

In this survey study using a DCE, a subset of parents prioritized in-person care for pediatric ARTI care, and most parents had other profiles focused on different care attributes. Results of this research suggest that access to low-cost, quickly available telehealth services with a clinician in a child’s usual practice could shift some urgent care and emergency department visits for ARTI to the child’s primary care practice.

## Key Points

**Question:**

What attributes of care sites do parents prefer when seeking care for a young child’s acute respiratory tract infection (ARTI)?

**Findings:**

In this survey study including 944 participants, respondents gave most weight to visit modality (in person vs telemedicine; importance, 22.7%) and out-of-pocket costs (23.7%) and least weight to noncare time (5.8%) and availability of follow-up within 2 days (6.8%).

**Meaning:**

Results suggest that access to low-cost, quickly available telehealth services with a clinician in a child’s usual practice could shift some urgent care and emergency department visits for ARTIs to the child’s primary care practice.

## Teaser

This survey study uses a discrete choice experiment to determine the importance parents assign to different attributes of sites of care for their child with a respiratory infection.

## Introduction

Telehealth is the use of electronic information and telecommunications technologies to extend care when a patient and clinician are not in the same place at the same time. Telehealth use expanded rapidly during the COVID-19 pandemic and has been touted to reduce patient travel time and costs and increase access options for patients while also decreasing transmission of infectious disease.^1,2,3^ Just as in-person care can vary across multiple dimensions (continuity, cost, timeliness), telehealth care can also vary in continuity (eg, with patient’s primary care practice vs virtual-only vendor), cost, and timeliness. Professional societies, such as the American Academy of Pediatrics,^4^ prioritize care within the patient-centered medical home when possible to improve care quality and enhance patient-clinician continuity.^5^ Because insurers currently cover primary care–delivered telehealth, medical homes must now consider whether and how to incorporate telehealth services into their practices.

For patients and families, the decision to use telehealth rather than attend an in-person visit is driven, in part, by patient preferences and perceptions of their options.^6,7,8,9,10^ Previous qualitative work by some of the authors of this study found that parents of young children consider attributes related to care sites (expected access, affordability, clinical quality, and site quality) and child or family factors (perceived illness severity, perceived child susceptibility, and parent self-efficacy) when choosing where to seek care for an acute respiratory tract infection (ARTI).^6^ This prior work did not assess the relative importance of these attributes in parents’ decision-making process.

Discrete choice experiments (DCEs) are a rigorous approach to understanding how individuals weigh attributes when making a particular decision. DCEs have been used extensively to inform the design of both consumer and health care products^11^ by measuring the tradeoffs that individuals are willing to make in a hypothetical decision.^12,13^ However, almost all DCE studies of virtual care choices have taken place outside the US, and none have focused on pediatric populations.

In this study, we quantify how parents of young children in the US weigh different attributes when deciding on pediatric care and examine the heterogeneity in these weights. We study this in the context of ARTIs, given the high prevalence of pediatric care seeking for ARTI symptoms.^1^ The results may improve our understanding of how parents select a care delivery method for their young child with an ARTI and how systems could optimize their telehealth care offerings to encourage selection of sites that promote care continuity and quality.

## Methods

### Study Design

This survey study uses DCE methods commonly applied to measure the relative value of competing health-related services in hypothetical scenarios.^12,13^ The DCE presents respondents with multiple choices and asks them to select the choice that they prefer. The respondent is asked to repeat this task with different choices multiple times within a survey. Using respondent selections over multiple tasks, it is possible to identify the attributes that considerably influence choices and evaluate the relative preference for different levels of these attributes. This study follows the Consolidated Health Economic Evaluation Reporting Standards (CHEERS) reporting guideline for DCE studies.^12,13,14^ The survey was determined exempt from human subjects review by the University of Pittsburgh Institutional Review Board (IRB) and the National Opinion Research Center (NORC) at the University of Chicago IRB (STUDY22120018) because it is a survey of adults that does not contain sensitive information. Informed consent was waived by the reviewing bodies. The University of Pittsburgh research team received deidentified data from NORC.

### Development of DCE Attributes and Levels

The DCE method requires careful selection of a specific decision scenario (eg, a care site decision), the decision’s attributes, and possible levels within each attribute. This DCE’s attributes are based on prior qualitative research.^6^ An initial draft of the scenario, attributes, and levels was developed by the authors with an emphasis on selecting attributes that a health delivery system could modify. This draft was iteratively refined by an expert advisory board including clinicians and health policy experts. We performed hour-long, 1-on-1 cognitive tests with parents of young children recruited through investigator networks and connections (n = 5). The final scenario was designed to create a sense of need for care (Box). The final set of attributes and levels used in the DCE is presented in Table 1.

Box. **Scenario Guidance**

**Final Design Scenario**

The purpose of this study is to get a better understanding of what is important to you when making decisions about where to go when your child is sick.
In this survey, we’ll ask you to imagine that your child is sick and to consider different options for where you could seek care for your sick child. Specifically, we will ask you to consider the following scenario:
  Imagine your child was doing well until 3 days ago when they developed a cough and runny nose. Yesterday, they developed a fever of 102 °F, and this morning they again have a fever of 102 °. You have done what you normally do at home for your child when they are sick, and it is not helping. They look sick to you and not like themselves in a way that makes you feel worried. You use a home COVID-19 test on your child and it is negative today. You decided they need to be seen by a health care provider for these symptoms.

**Table 1.** Attributes and Levels Used in the DCE^a^

Description to participant	Attribute label	Levels as described to participants
This is where and how the visit happens, and how you and your child connect with the health care provider.	Visit type	In-person visit (such as at a primary care practice, urgent care center, emergency department, or hospital) Live audio and/or video virtual visit at home (through your personal device) Live audio and/or video virtual visit at a telemedicine site (eg, at a pharmacy, library, school, or shopping center)
This is how soon the appointment will occur.	Appointment timing	In 30 min In 3 h In 6 h Tomorrow
This is the time you expect to spend scheduling, registering, waiting after check-in, checking out, and paying. This does not include the time actually spent receiving care.	Noncare time	~ 5 min ~ 15 min ~ 30 min ~ 60 min
This is the amount you expect to pay personally. This does not include the costs your insurance pays.	Costs to you	$0 $10 $20 $40 $80
This is whether you see a health care provider who knows your child and/or has access to your child’s records during the visit.	Continuity	With your child’s usual health care provider who has access to their medical records With any health care provider in your child’s usual group or practice who has access to their medical records Outside of your child’s usual group or practice who has access to their medical records Outside of your child’s usual group or practice who does not have access to their medical records
This is whether you receive care from a health care provider who has training and experience in caring for children.	Child focus	With a health care provider who regularly cares for children (such as pediatrics or family medicine) With a provider who does not regularly care for children
This is whether the same health care provider your child sees today is able to see your child again for the same illness in the next 2 d if they don’t start to get better.	Potential for follow-up	Available for follow-up in 2 d Not available for follow-up

Abbreviation: DCE, discrete choice experiment.

^a^ Using exact terminology from the questions to which respondents addressed.

### Design of DCE

We use a full-profile design that uses random sampling with replacement with Sawtooth software^15^ (Sawtooth Software, Inc). The software generates choice pairs using the predefined attributes and levels (eg, choice 1 and choice 2). Selection of the choice pairs was performed with Sawtooth’s balanced overlap design. Respondents were asked to select their preference between choice 1 and choice 2. Respondents were offered a third choice stating, “I would not seek care if these were my only options.” Sample size estimates were based on the numbers of attributes, levels, and choice tasks. With 12 random choice pairs per respondent, we had a target sample size of 325 respondents for the primary analysis.^16,17^

### Study Sample

Nationally representative study participants were recruited from the AmeriSpeak panel^18^ maintained by NORC at the University of Chicago. AmeriSpeak participants who listed children younger than 12 years in their household in their panel information were invited to participate in this study between July 31 and August 18, 2023. Potential respondents were then screened to confirm that they were involved in medical decision making for at least 1 child currently between the ages of 6 months and 5 years. The survey was conducted in both English and Spanish through a web browser. Survey respondents were compensated through AmeriSpeak, receiving a $5 cash equivalent.

### Administration of DCE Survey

Respondents viewed the scenario and attributes and then completed 12 choice tasks. After the choice tasks, respondents completed questions about health care insurance and access, their child’s health and health care use, digital health access, digital health literacy, work schedules, and languages spoken at home. Self-identified race was collected because respondent race, a social construct, can affect access to and experiences with medical care.

### Statistical Analysis

For analysis of the DCE, the dependent variable was the respondent’s choice in each choice task and the independent variables were the attribute levels.^19^ We performed choice-based conjoint analysis using Sawtooth Software’s Lighthouse Studio 9.15.1. We used 2 analysis models: a hierarchical bayesian (HB) model and a weighted latent class logistic model. HB modeling allows for individual preference heterogeneity and all individuals to have different preferences. All parameters were specified as normally distributed. We used person-weights, which were developed by NORC to account for panel sampling weights as well as survey response rates when creating summary statistics from the HB model.

We report part-worth (PW) utilities for each level of each attribute; PW utilities represent the desirability of each attribute level relative to each other level. These utilities are zero-centered unitless measures in which a larger PW indicates a stronger preference. A positive PW indicates a positive preference, and a negative PW indicates a negative preference. The importance score of an attribute is calculated by comparing the PWs of the most and least influential attribute levels. The difference between these weights is summed across all attributes and scaled to 100. The importance of each attribute is then expressed as a percentage of this total.

We used latent class analysis to detect heterogeneity in group preference patterns.^20^ To guide the selection of the number of latent classes, we compared model fit statistics for solutions with 2 to 5 groups including Akaike and bayesian information criteria and relative χ^2^ difference. We also considered the size of each group and the interpretability and distinctness of the resulting groups. The latent class model does not assign respondents to a class but does generate membership probabilities for each respondent. We assigned each respondent to the class with their highest membership probability. We then reported descriptive statistics of each latent class.

We performed a market simulation based on the latent class utilities to compare shares of preferences in 2 simulations. In simulation 1, we simulated parent choice of care site when given 4 typical options: an in-person visit with their child’s regular clinician, an urgent care visit, an emergency department visit, or no care. Simulation 2 added an additional care option that could be provided by a health system: a telemedicine visit with a clinician in the child’s usual practice group. For each of the options, we set levels for each attribute through consensus with the investigator team, which included 5 clinician researchers and 4 scientists of various disciplines. The attribute levels of each care option are in Table 1. Shares of preferences for each option in simulation 1 (4 options) and simulation 2 (5 options) were estimated using a randomized first-choice simulation method.

## Results

AmeriSpeak extended invitations to 8952 panelists, and 28% of invited panelists completed the screening. Of those, 40% were found eligible, and 95% of those eligible completed the survey. The final sample consisted of 944 parents from all 50 states and the District of Columbia. Among the sample, 120 (12.7%) identified as non-Hispanic Black and 190 (20.1%) as Hispanic. Three hundred thirty-eight (35.8%) respondents indicated that their children were Medicaid beneficiaries, and 285 (30.2%) reported speaking a language other than English at home (eTable 1 in Supplement 1).

**Figure 1.**
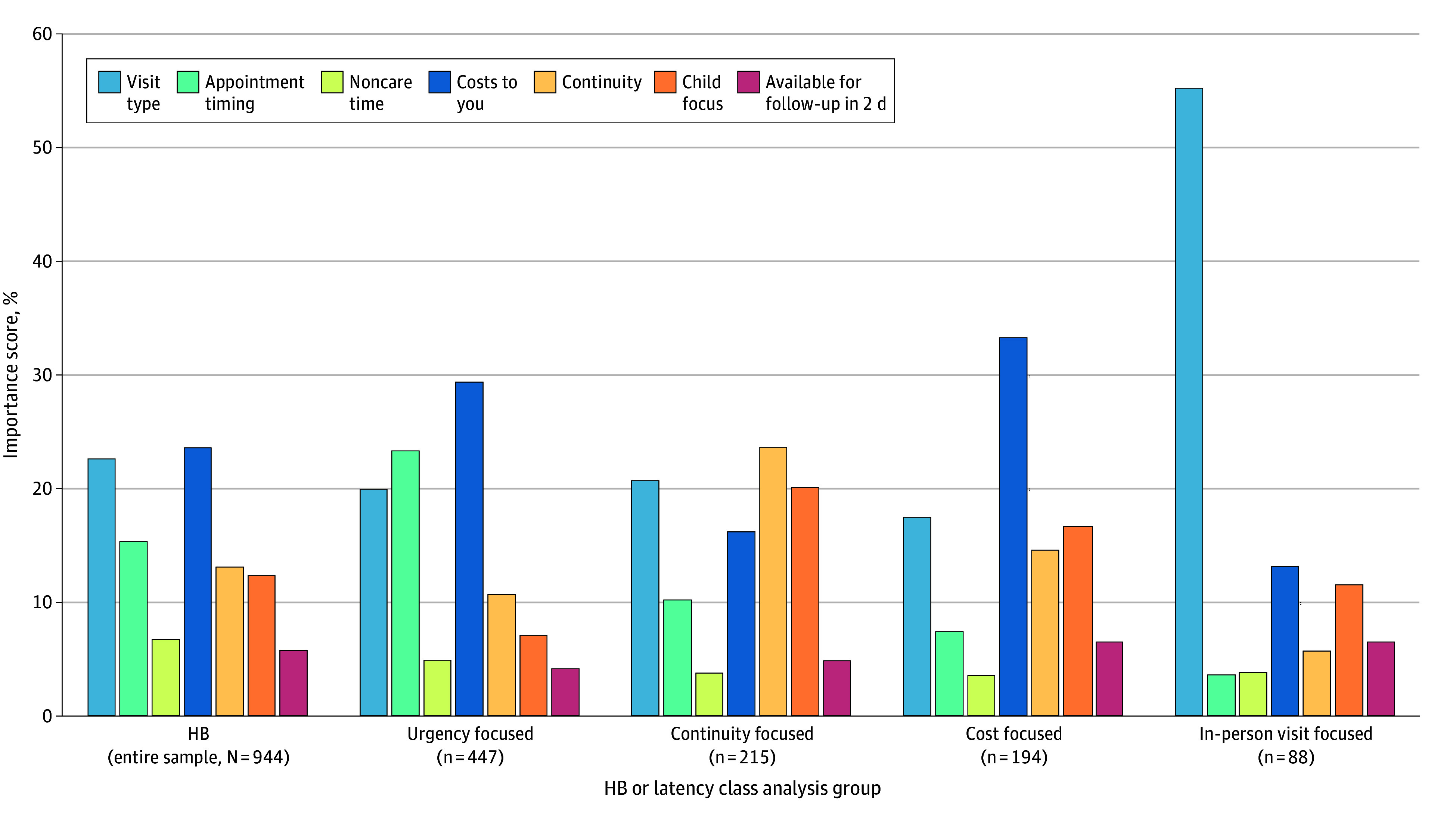
Importance Scores Histogram for Hierarchical Bayesian (HB) Analysis and 4 Latent Classes

In the HB analysis, the strongest attribute level preferences within the full sample were avoiding the highest cost and having an in-person visit (Table 2; eFigure 2A in Supplement 1). The most important attributes were costs (23.7%) and visit type (22.7%; Figure 1). The least important attributes were noncare time (6.8%) and availability of follow-up (5.8%). The PW utility not seek care provides the estimated threshold at which a respondent would decline a care setting. For the HB analysis of the full sample, this threshold was −123.7 (Table 2), indicating that a care option would be rejected by the average respondent only if the sum of its PW attribute utilities was less than −123.7.

**Table 2.** PW UEs and IS for the HB and Latent Class Analyses

Characteristic	Analysis model				Latent class analysis, focus							
	HB		Latent class		Urgency		Continuity		Cost		In-person visit	
	PW UE (95% CI) (N=944)	IS^a^	PW UE (SE) (N=944)	IS^a^	PW UE (SE) (n=447)	IS^a^	PW UE (SE) (n=215)	IS^a^	PW UE (SE) (n=194)	IS^a^	PW UE (SE) (N=88)	IS^a^
Visit type		23		23		20		21		18		55
In person	71 (66.17-75.82)		85 (1.54)		71 (0.27)		74 (0.37)		62 (0.70)		234 (2.78)	
Video virtual visit at home	−9 (−11.61 to −6.88)		−9 (0.62)		−1 (0.09)		−2 (0.12)		−1 (0.28)		−81 (1.12)	
Virtual visit at a telemedicine site	−62 (−65.18 to −58.32)		−76 (0.94)		−70 (0.19)		−72 (0.29)		−61 (0.43)		−153 (1.66)	
Appointment timing		15		15		23		10		8		4
In 30 min	35 (33.23-37.57)		47 (0.12)		68 (0.02)		31 (0.03)		33 (0.05)		11 (0.17)	
In 3 h	15 (13.34-16.41)		21 (0.13)		35 (0.04)		14 (0.06)		0 (0.12)		12 (0.14)	
In 6 h	−1 (−2.07 to 0.6)		−8 (0.10)		−6 (0.02)		−3 (0.04)		−16 (0.06)		−11 (0.08)	
Tomorrow	−50 (−53.27 to −45.82)		−60 (0.30)		−96 (0.07)		−41 (0.12)		−17 (0.19)		−12 (0.22)	
Noncare time, min		7		4		5		4		4		4
~ 5	3 (1.87-4.31)		8 (0.06)		11 (0.01)		3 (0.02)		8 (0.04)		−4 (0.12)	
~ 15	6 (4.92-7.5)		7 (0.06)		5 (0.02)		7 (0.03)		11 (0.03)		7 (0.04)	
~ 30	3 (1.6-3.69)		6 (0.11)		7 (0.03)		8 (0.05)		−5 (0.08)		12 (0.18)	
~ 60	−12 (−13.52 to −10.36)		−20 (0.08)		−23 (0.03)		−18 (0.05)		−15 (0.04)		−15 (0.09)	
Costs to you, $		24		26		29		16		33		13
0	52 (48.68-54.87)		65 (0.24)		78 (0.06)		33 (0.11)		83 (0.18)		39 (0.17)	
10	38 (35.78-40.19)		47 (0.20)		55 (0.05)		23 (0.10)		63 (0.16)		27 (0.15)	
20	22 (21.07-23.76)		27 (0.10)		32 (0.04)		25 (0.07)		27 (0.04)		10 (0.08)	
40	−19 (−20.96 to −16.85)		−24 (0.18)		−37 (0.05)		0 (0.12)		−22 (0.07)		−22 (0.11)	
80	−93 (−97.43 to −89.10)		−115 (0.46)		−128 (0.14)		−81 (0.19)		−151 (0.33)		−54 (0.46)	
Continuity		13		14		11		24		15		6
Usual clinician with records	31 (28.67-32.36)		42 (0.56)		31 (0.22)		81 (0.44)		33 (0.25)		22 (0.12)	
Usual group with records	27 (24.72-28.29)		31 (0.44)		27 (0.13)		52 (0.26)		40 (0.19)		−16 (0.51)	
Outside group with records	−15 (−16.39 to −13.29)		−20 (0.37)		−13 (0.13)		−47 (0.28)		−12 (0.17)		0 (0.11)	
Outside group without records	−42 (−44.39 to −39.97)		−54 (0.59)		−44 (0.22)		−85 (0.42)		−61 (0.24)		−5 (0.38)	
Child focus		12		13		7		20		17		12
Yes	41 (38.73-42.47)		44 (0.50)		25 (0.21)		71 (0.36)		59 (0.19)		41 (0.27)	
No	−41 (−42.47 to −38.73)		−44 (0.50)		−25 (0.21)		−71 (0.36)		−59 (0.19)		−41 (0.27)	
Available for follow-up in 2 d		6		5		4		5		7		7
Yes	15 (13.33-16.04)		18 (0.14)		15 (0.04)		17 (0.06)		23 (0.06)		23 (0.17)	
No	−15 (−16.04 to −13.33)		−18 (0.14)		−15 (0.04)		−17 (0.06)		−23 (0.06)		−23 (0.17)	
Neither of the available options	−124 (−134.85 to −112.51)		−248 (2.66)		−500 (0.71)		−144 (0.98)		77 (1.54)		65 (2.17)	

Abbreviations: HB, hierarchical bayesian; PW UE, part-weight utility estimate; IS, importance score.

^a^ The importance score of an attribute is calculated by comparing the PWs of the most and least influential attribute levels. The difference between these weights is summed across all attributes and scaled to 100. The importance of each attribute is then expressed as a percentage of this total.

Latent class analysis identified 4 profiles: urgency focused, continuity focused, cost focused, and in-person visit focused (Table 2). The urgency-focused group (47.4% of respondents) had the lowest threshold to accept care (PW of none, −500.2) of any group, indicating that they would accept a suboptimal care option even if they viewed the attribute levels to be less preferable (Table 2). Compared with other groups, they had the strongest negative PW, indicating the least preference, for the attribute level of an appointment tomorrow (eFigure 2B in Supplement 1). For this group, the type of care selected was sensitive to out-of-pocket costs (importance, 29.5%), timeliness of appointment (23.4%), and visit modality (20.0%; Figure 1). Group members were more likely to report Hispanic ethnicity, more difficulty getting to medical care, lower health literacy, and lower English proficiency (eTable 1 in Supplement 1). This group was least likely to report prior in-person primary care visits for a sick child (82%; eTable 2 in Supplement 1).

The continuity-focused group (22.8% of respondents) was most sensitive to preserving continuity of care (importance, 23.7%), soliciting pediatric expertise (20.2%), and visit modality (20.8%; Table 2). Group members were more likely to identify as non-Hispanic White and have a college or graduate degree, income over $100 000, employer-based health insurance, younger children, high health literacy, and high English proficiency (eTable 1 in Supplement 1).

The cost-focused group (20.6% of respondents) had the highest threshold to accept care (PW of none, 77.0), meaning they would choose to reject all but the most optimal options, unlike the other groups (Table 2). This group was sensitive to cost (importance, 33.4%). Group members were more likely to identify as Black and report high school as their highest level of education, income less than $30 000, and receipt of government-based insurance for their children (eTable 1 in Supplement 1). Group members were most likely to report never having sought sick care for their children compared with other groups (8.1%; eTable 2 in the Supplement).

The in-person visit–focused group (9.3% of respondents) was most sensitive to visit modality (importance, 55.3%; Table 2). Group members were more likely to identify as non-Hispanic White and report high health literacy (eTable 1 in Supplement 1). This group was most likely to report prior use of primary care for a sick child (95.8%; eTable 2 in Supplement 1).

In the market simulations (Table 3), compared with the scenario including the options of primary care, emergency department care, and urgent care (simulation 1), the addition of a quickly available, low-cost telemedicine option with a practitioner in the child’s usual practice group (simulation 2) shifted the preferred site of care for an ARTI for all latent classes except the in-person visit–focused group. For the continuity-focused group, 25.3% of simulated choices switched from the in-person primary care physician (PCP) visit option to the PCP telemedicine option without changes to other care sites. For the urgency-focused group, simulated preferred care sites changed such that urgent care visits were reduced from 26.4% to 17.8%, emergency department visits were reduced from 16.8% to 12.1%, and choice of primary care combined (telemedicine or in person) increased from 55.8% to 69.6%. For the cost-focused group, preferred care sites also changed, with a decrease from 8.2% to 4.4% for urgent care visits and a decrease from 4.6% to 2.7% for emergency department visits. For the cost-focused group, simulation 2 yielded a reduction in the number of people who would not seek any care (21.4% to 13.3%) and an increase from 65.9% to 79.5% in the choice of either of the primary care options.

**Table 3.** Care Decisions by Latent Class for Current Landscape and Current Landscape With Hypothetical Usual Practice Telemedicine Visit

Variable	Usual PCP in-person visit	Urgent care visit	Emergency department visit	Usual primary care practice telemedicine visit	Decline to seek care
Description					
Visit type	In person	In person	In person	At home	NA
Appointment timing	3 h	30 min	3 h	30 min	NA
Noncare time	30 min	30 min	60 min	15 min	NA
Costs to you	$20	$40	$80	$20	NA
Continuity	Usual clinician with records	Outside group without records	Outside group without records	Usual group with records	NA
Child-focused provider	Yes	No	No	Yes	NA
Follow-up available	Yes	No	No	Yes	NA
Simulation 1: current landscape without usual practice telemedicine, %					
Urgency focused	55.8	26.4	16.8	NA	1.0
Continuity focused	96.1	2.1	0.9	NA	0.9
Cost focused	65.9	8.2	4.6	NA	21.4
In-person visit focused	82.7	9.8	5.9	NA	1.6
Simulation 2: current landscape plus usual practice telemedicine, %					
Urgency focused	37.3	17.8	12.1	32.3	0.6
Continuity focused	70.8	1.0	0.5	27.3	0.4
Cost focused	41.1	4.4	2.7	38.4	13.3
In-person visit focused	82.9	9.6	5.5	0.4	1.5
Change in care location (simulation 2 − simulation 1) with usual practice telemedicine addition, %					
Urgency focused	−18.5	−8.6	−4.7	32.3	−0.5
Continuity focused	−25.3	−1.1	−0.5	27.3	−0.5
Cost focused	−24.8	−3.7	−1.8	38.4	−8.1
In-person visit focused	0.2	−0.2	−0.4	0.4	−0.1

Abbreviation: NA, not applicable; PCP, primary care physician.

## Discussion

This study evaluates the preferences of parents of young children for site of care when their child has ARTI symptoms. We found 4 distinct latent classes of care site preferences that focused on urgency, continuity, cost, and visit modality, with wide variation in the threshold for seeking care in each group. Each of these latent classes responded differently in simulations of care site choices when a primary care telemedicine option was added to a scenario with in-person PCP visit, urgent care, and emergency department care. When this primary care telemedicine option was added, there was a substantial shift in simulated preferred care site for the urgency-focused and cost-focused groups, with a larger percentage anticipated to prefer care within their primary care practice (vs urgent care, emergency department care, or no care) when primary care telemedicine was offered. In contrast, no net change in simulated preferences for primary care options occurred for the in-person care–focused group, and only minimal shifts occurred for the continuity-focused group; both groups already seemed highly connected to primary care.

The results of our simulation analysis highlight that well-designed primary care telemedicine options may be associated with access to and reduce fragmentation of care by meeting the need for timely, low-cost care through virtual visits while still offering in-person care to those patients for whom in-person care is a priority. Because the cost-focused and urgency-focused groups identified in our latent class analysis had greater representation of individuals from racial and ethnic minoritized backgrounds, individuals with children insured by Medicaid, and individuals with potential language and literacy barriers, these results suggest that primary care telemedicine could be a targeted strategy to reduce disparities in the use of primary care in these populations. However, the access barriers (language, literacy) and priorities (urgency, costs) of these populations should be proactively incorporated into the design of such telemedicine programs.

To our knowledge, there are no other DCEs that have evaluated preferences for telehealth in US pediatric populations, although there are a few studies that have evaluated parents’ preferences for care site attributes. One US study found that parents selecting a primary care practice had preferences that were strongest for access to same-day sick visits followed by continuity with their child’s PCP.^21^ A 2023 study conducted in Ireland found that parents’ decisions about accessing unscheduled health care were most sensitive to cost, same-day or next-day access, and care from their usual practitioner.^22^ However, neither study included an analysis of latent classes or virtual options. We know of only 2 other latent class evaluations in telemedicine DCE studies, which found 4 and 5 latent classes.^23^ Neither focused on pediatric care (or on the attributes identified in the prior pediatric-focused qualitative work by some of the authors of this study^6^), but both found a class with a strong preference for face-to-face appointments, and 1 found a class responding strongly to cost.

### Limitations

The responses in this study were all collected within the US context of health care access and costs, so results are unlikely to be informative in other global contexts. Likewise, this study uses an online panel and only collected data in English and Spanish, so generalizability to the entire US population may be limited. DCEs also have several inherent limitations. First, DCE results are only as useful as the attributes and levels used to describe the service options. We informed our selection of attributes and levels by qualitative and quantitative research and focused on deliberate, modifiable attributes. Adding more attributes and levels may have provided more information but could have counterproductively made the experiment more cognitively demanding for respondents and thus less informative.^24^ Also, the relative importance of an attribute can be influenced by the range of total attributes presented, so the importance given to each attribute by respondents must be viewed within the context of the particular set of attributes and levels used in this study. Another major limitation with DCEs is that they use stated preferences that may differ from the revealed preferences (ie, actual decisions) of parents of children with ARTIs, particularly in the context of real-world constraints such as childcare access, work schedules, and transportation options. Additionally, this study focused on care-seeking preferences in a scenario in which we highlighted that the parent was feeling very concerned about their young child. It is unclear if the same latent classes or site preferences would be revealed with a different health condition, different severity of symptoms, or different age group.

## Conclusions

In this survey study using a DCE, we found 4 distinct classes of preferences for site of care among parents of young children with ARTIs. These distinct classes responded differently to simulated scenarios in which they could choose between different care options. Because the cost-focused and urgency-focused groups had greater representation of individuals from racial and ethnic minoritized backgrounds, primary care telemedicine could provide a path to address equity concerns about primary care access.

## Notes

Supplement 1. **eFigure 1.** Screenshot of Example Choice Task **eFigure 2.** Part-Worth Utility Values for All Attributes and Levels **eTable 1.** Sample Characteristics in the Overall Sample and by Latent Class, With Sample Weights **eTable 2.** Prior Acute Care Utilization in the Overall Sample and by Latent Class, With Sample Weights

Supplement 2.
Data Sharing Statement

## References

[1] Schweiberger K, Patel SY, Mehrotra A, Ray KN. Trends in pediatric primary care visits during the coronavirus disease of 2019 pandemic. Acad Pediatr. 2021;21(8):1426-1433. doi:10.1016/j.acap.2021.04.031 33984496 PMC8561008

[2] Schweiberger K, Hoberman A, Iagnemma J, . Practice-level variation in telemedicine use in a pediatric primary care network during the COVID-19 pandemic: retrospective analysis and survey study. J Med Internet Res. 2020;22(12):e24345. doi:10.2196/24345 33290244 PMC7752181

[3] Health Resources and Services Administration. Telehealth policy updates. March 20, 2025. Accessed June 16, 2025. https://telehealth.hhs.gov/providers/telehealth-policy/telehealth-policy-updates

[4] Katz SE, Spencer P, Stroebel C, Harnack L, Kastner J, Banerjee R. Patient and provider perspectives on pediatric telemedicine during the COVID-19 pandemic. Telemed Rep. 2021;2(1):293-297. doi:10.1089/tmr.2021.0032 35720742 PMC9049794

[5] Conners GP, Kressly SJ, Perrin JM, Richerson JE, Sankrithi UM; Committee on Practice and Ambulatory Medicine; Committee on Pediatric Emergency Medicine; Section on Telehealth Care; Section on Emergency Medicine; Subcommittee on Urgent Care; Task Force on Pediatric Practice Change. Nonemergency acute care: when it’s not the medical home. Pediatrics. 2017;139(5):e20170629. doi:10.1542/peds.2017-0629 28557775

[6] Burns SK, Krishnamurti T, Doan TT, Kahn JM, Ray KN. Parent care-seeking decisions for pediatric acute respiratory tract infections in the US: a mental models approach. Acad Pediatr. 2023;23(7):1326-1336. doi:10.1016/j.acap.2023.02.011 36871609 PMC10475487

[7] Morgan JW, Salmon MK, Ambady M, . Factors informing high-risk primary care patient choice around telehealth use: a framework. J Gen Intern Med. 2024;39(4):540-548. doi:10.1007/s11606-023-08491-y 37940757 PMC10973282

[8] Nicholson E, McDonnell T, De Brún A, . Factors that influence family and parental preferences and decision making for unscheduled paediatric healthcare - systematic review. BMC Health Serv Res. 2020;20(1):663. doi:10.1186/s12913-020-05527-5 32680518 PMC7366445

[9] Uscher-Pines L, Pines J, Kellermann A, Gillen E, Mehrotra A. Emergency department visits for nonurgent conditions: systematic literature review. Am J Manag Care. 2013;19(1):47-59. 23379744 PMC4156292

[10] Turnbull J, McKenna G, Prichard J, . Sense-making strategies and help-seeking behaviours associated with urgent care services: a mixed-methods study. Health Services and Delivery Research. 2019;7(26). doi:10.3310/hsdr07260 31356036

[11] van den Broek-Altenburg E, Atherly A. Using discrete choice experiments to measure preferences for hard to observe choice attributes to inform health policy decisions. Health Econ Rev. 2020;10(1):18. doi:10.1186/s13561-020-00276-x 32529586 PMC7291477

[12] Hauber AB, González JM, Groothuis-Oudshoorn CG, . Statistical methods for the analysis of discrete choice experiments: a report of the ISPOR Conjoint Analysis Good Research Practices Task Force. Value Health. 2016;19(4):300-315. doi:10.1016/j.jval.2016.04.004 27325321

[13] Reed Johnson F, Lancsar E, Marshall D, . Constructing experimental designs for discrete-choice experiments: report of the ISPOR Conjoint Analysis Experimental Design Good Research Practices Task Force. Value Health. 2013;16(1):3-13. doi:10.1016/j.jval.2012.08.2223 23337210

[14] Bridges JF, Hauber AB, Marshall D, . Conjoint analysis applications in health–a checklist: a report of the ISPOR Good Research Practices for Conjoint Analysis Task Force. Value Health. 2011;14(4):403-413. doi:10.1016/j.jval.2010.11.013 21669364

[15] Sawtooth Software, Inc. Choice-based conjoint analysis. 2017. Updated 2021. Accessed November 7, 2023. https://sawtoothsoftware.com/conjoint-analysis/cbc

[16] Orme B. Sample size issues for conjoint analysis. 2019. Accessed June 16, 2025. https://content.sawtoothsoftware.com/assets/dd3f6a38-285f-441f-a88c-678d7c8aaffb

[17] Johnson R, Orme B. Getting the most from CBC. 2003. Accessed June 16, 2025. https://sawtoothsoftware.com/resources/technical-papers/getting-the-most-from-cbc

[18] National Opinion Research Center. AmeriSpeak panel design. Accessed April 14, 2023. https://amerispeak.norc.org/us/en/amerispeak/about-amerispeak/panel-design.html

[19] Huber J, Orme B, Miller R. The Value of Choice Simulators. Conjoint Measurement. 4th ed. Springer; 2007.

[20] Sawtooth Software, Inc. The latent class technical paper v4.8. 2021. Accessed June 16, 2025. https://sawtoothsoftware.com/resources/technical-papers/latent-class-technical-paper

[21] Zickafoose JS, DeCamp LR, Prosser LA. Parents’ preferences for enhanced access in the pediatric medical home: a discrete choice experiment. JAMA Pediatr. 2015;169(4):358-364. doi:10.1001/jamapediatrics.2014.3534 25643000 PMC4545238

[22] Nicholson E, McDonnell T, Conlon C, De Brún A, Doherty E, McAuliffe E. Parent’s preferences for unscheduled paediatric healthcare: a discrete choice experiment. Health Expect. 2023;26(5):1931-1940. doi:10.1111/hex.13802 37338038 PMC10485340

[23] Buchanan J, Roope LSJ, Morrell L, . Preferences for medical consultations from online providers: evidence from a discrete choice experiment in the United Kingdom. Appl Health Econ Health Policy. 2021;19(4):521-535. doi:10.1007/s40258-021-00642-8 33682065 PMC7937442

[24] Bech M, Kjaer T, Lauridsen J. Does the number of choice sets matter? Results from a web survey applying a discrete choice experiment. Health Econ. 2011;20(3):273-286. doi:10.1002/hec.1587 20143304

