# Supplementary material for: Parent Preferences for Acute Respiratory Tract Infection Care
Source: JAMA Netw Open. 2025 Aug 8;8(8):e2525904. doi: 10.1001/jamanetworkopen.2025.25904 (PMC12334952; doi:10.1001/jamanetworkopen.2025.25904)
Supplement: Supplement 1. — eFigure 1. Screenshot of Example Choice Task eFigure 2. Part-Worth Utility Values for All Attributes and Levels eTable 1. Sample Characteristics in the Overall Sample and by Latent Class, With Sample Weights eTable 2. Prior Acute Care Utilization in the Overall Sample and by Latent Class, With Sample Weights [file jamanetwopen-e2525904-s001.pdf]

Supplemental Online Content

Hanmer J, Burns SK, Wittman SR, Doan TT, Krishnamurti T, Ray KN. Parent preferences for acute respiratory tract infection care. *JAMA Netw Open*. 2025;8(8):e2525904. doi:10.1001/jamanetworkopen.2025.25904

eFigure 1. Screenshot of Example Choice Task

eFigure 2. Part Worth Utility Values for All Attributes And Levels, Hierarchical Bayesian Analysis and Latent Class Analysis

eTable 1. Sample Characteristics in the Overall Sample and by Latent Class, With Sample Weights

eTable 2. Prior Acute Care Utilization in the Overall Sample and by Latent Class, With Sample Weights

This supplemental material has been provided by the authors to give readers additional information about their work.

© 2025 Hanmer J et al. *JAMA Network Open*.

Supplemental eFigure 1: Screenshot of Example Choice Task.

Which of these options would you choose?

(3 of 12)

**Visit Type**	In-person visit (such as at a primary care practice, urgent care center, emergency department, or hospital)	Live audio/video virtual visit at home (through your personal device)	
**Appointment Timing**	Tomorrow	In 6 hours	
**Non-care time**	About 15 minutes	About 60 minutes	
**Costs to you**	$40	$0	
**Continuity**	Outside of your child's usual group/practice and has access to their medical records	Outside of your child's usual group/practice and has access to their medical records	I would not seek care if these were my only options
**Child Focus**	With a provider who does NOT regularly care for children	With a provider who does NOT regularly care for children	
**Available for Follow-up in Two Days**	Available for follow-up	NOT available for follow-up	
	Select	Select	Select

© 2025 Hanmer J et al. *JAMA Network Open*.

Supplemental eFigure 2A: Part worth utility values for all attributes and levels

Panel A: Hierarchical Bayesian Analysis

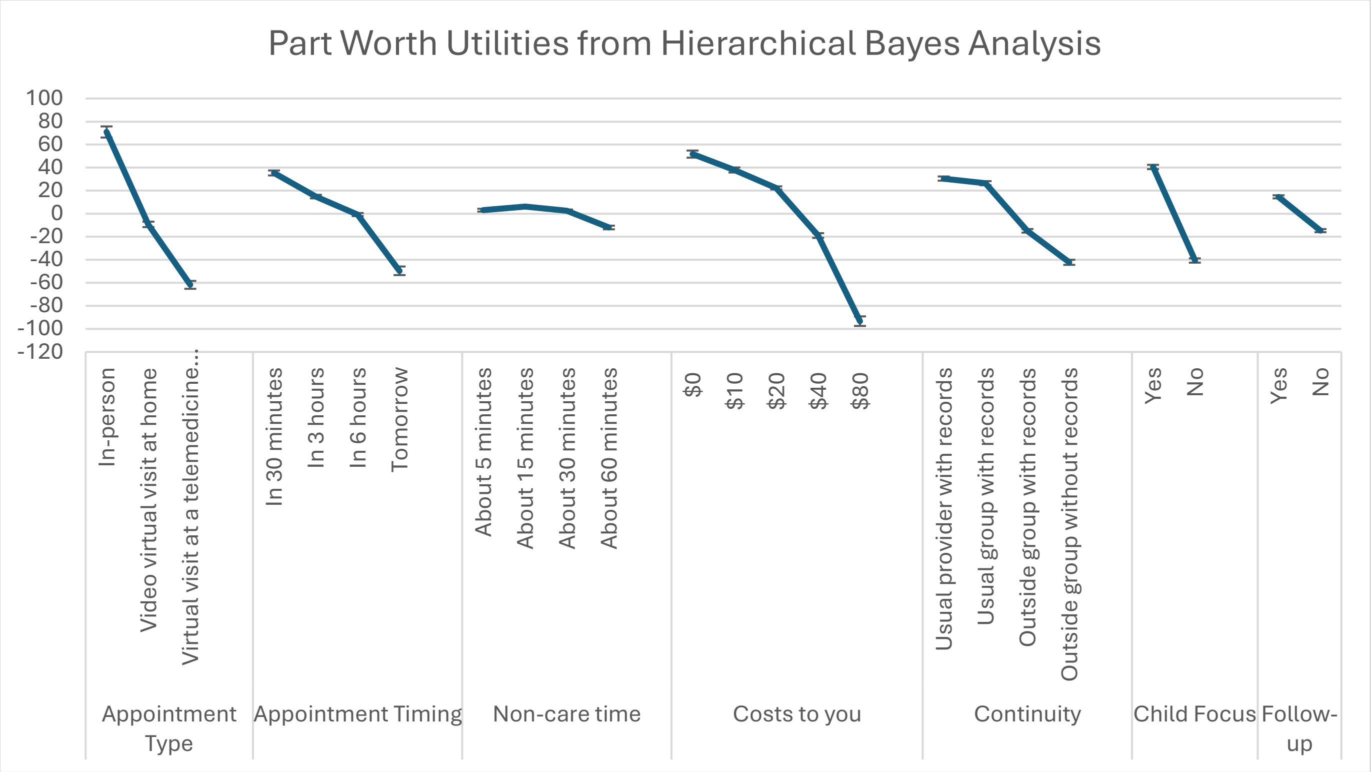

Panel B: Latent Class Analysis

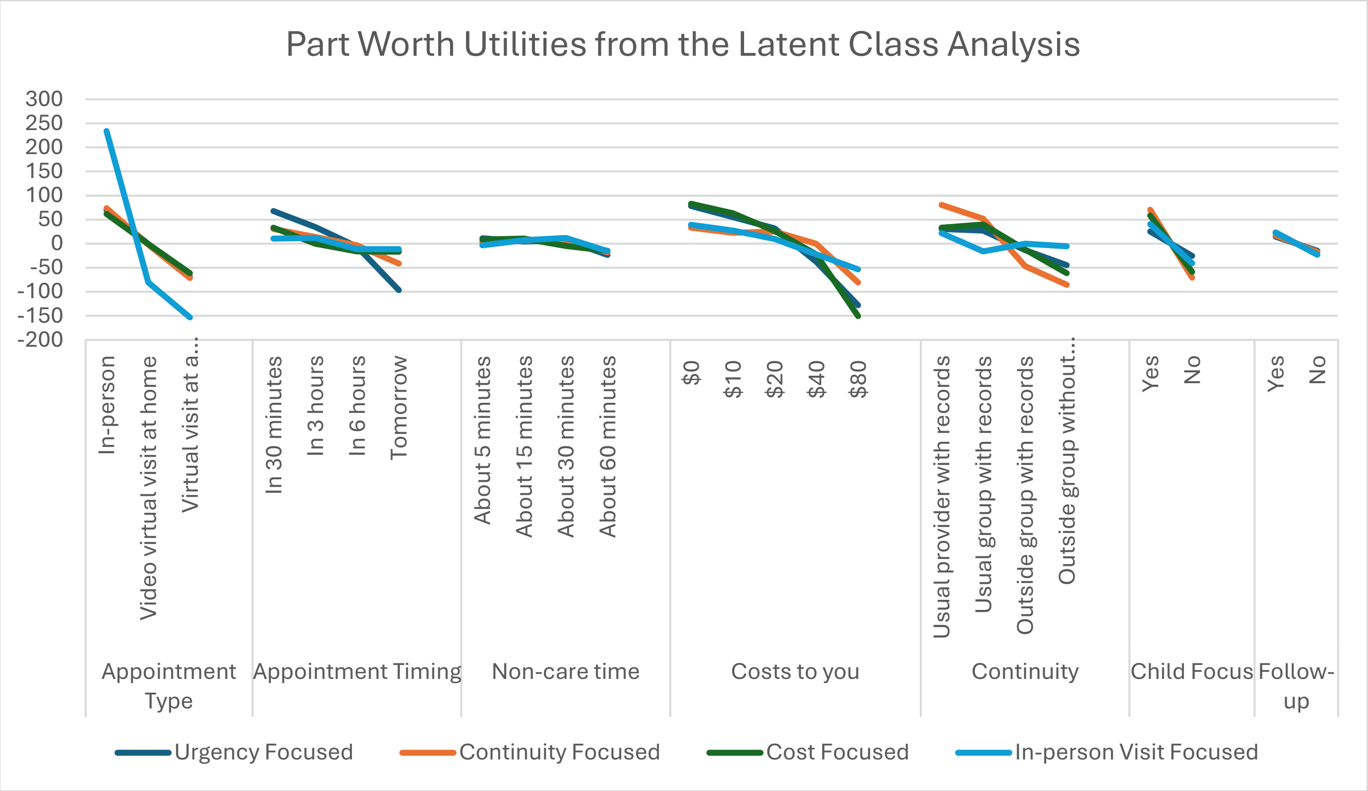

© 2025 Hanmer J et al. *JAMA Network Open*.

Supplemental eTable 1. Sample characteristics in the overall sample and by latent class, with sample weights.

	Whole Sample	Urgency focused	Continuity Focused	Cost Focused	In-person Visit Focused	Statistical testing (Pearson chi2 statistics with Rao&Scott correction for survey design)
**Weighted respondents**	944	475.9	208.7	181.9	77.4	
**Respondent Age (%)**						
18-29	32.1	32.0	3.04	36.3	18.0	p=0.30
30-44	64.6	64.6	62.7	61.4	77.7	
45+	3.3	3.4	3.3	2.3	4.3	
**Race/Ethnicity (%)**						
Asian-Pacific Islander	6.7	6.3	9.4	4.3	7.4	
Hispanic	24.2	27.4	20.5	22.9	17.0	
Black, non-Hispanic	12.8	14.2	5.8	19.1	8.3	
White, non-Hispanic	53.1	49.0	62.5	48.3	63.7	p=0.005
Other, non-Hispanic (including 2+ non-Hispanic)	3.3	3.0	1.8	5.5	3.6	
**Education (%)**						
HS or less	36.3	38.9	24.0	44.8	33.3	p=0.039
Some college/ associate's	20.3	16.5	23.8	24.1	25.9	
Bachelor's degree	25.0	25.9	39.6	18.1	23.3	
Post grad study/professional deg	18.4	18.8	22.6	13.1	17.2	
**Employment (%)**						
Working - as a paid employee	65.6	68.8	66.4	56.5	64.4	p=0.27

© 2025 Hanmer J et al. *JAMA Network Open*.

Working - self-employed	8.0	8.8	6.1	9.5	5.0	
Not working	26.4	22.3	27.6	34.0	30.5	
**Income (%)**						
Less than $30,000	25.7	26.2	18.4	32.9	25.4	p=0.027
$30,000 to under $60,000	26.9	26.8	26.4	27.7	27.7	
$60,000 to under $100,000	20.7	21.6	15.7	24.4	19.9	
$100,000 or more	26.6	25.3	49.5	15.0	27.0	
**Marital Status (%)**						
Married	66.3	67.1	69.5	57.2	74.4	p=0.12
Widowed, Divorced or Separated	5.3	7.0	2.6	4.7	4.4	
Never married	28.3	25.9	27.9	38.1	21.2	
**Metro (%)**						
Non-Metro	15.3	12.7	17.5	15.2	26.3	p=0.16
Metro	84.7	87.3	82.6	84.8	73.7	
**Age of Oldest Child (%)**						
6 months-2 years	4.6	3.0	8.6	5.2	2.1	p=<0.001
2-5 years	39.9	37.7	55.4	31.0	32.2	
6-10 years	31.2	32.5	23.1	35.6	34.4	
11-17 years	24.3	26.7	12.9	28.3	31.3	
**Insurance type (%)**						
Employer/Purchase Directly	53.1	50.6	65.5	41.1	63.8	p=0.006
Government	42.6	43.9	33.3	52.5	36.2	
Uninsured	4.3	5.6	1.2	6.4	0.0	
**Child with chronic condition (%)**						
Yes	15.1	14.8	14.8	13.6	21.9	p=0.63
**Usual source of care (%)**						
Yes	87.7	86.2	89.8	84.6	98.9	p=0.07
**Usual provider (%)**						

© 2025 Hanmer J et al. *JAMA Network Open*.

Yes	82.0	80.4	84.2	78.2	94.6	p=0.08
**Transportation barrier to medical appointment (%)**						
Yes	13.8	15.7	8.0	16.3	11.3	p=0.25
**Difficulty getting to medical care (%)**						
A little/not difficult	84.9	89.6	92.1	88.3	89.5	p=0.01
Somewhat or Very difficult	15.1	20.4	7.9	11.7	10.5	
**Health Literacy (%)**						
Somewhat/a little bit/not at all confident	21.0	27.7	12.6	18.4	8.0	p=0.005
Extremely/Quite confident	79.0	72.3	87.4	81.6	92.0	
**Understand English (%)**						
Very well	85.6	80.5	95.0	84.2	94.9	p=<0.001
Not at all, not well, or well	14.2	19.4	4.5	15.5	5.1	
**Speak language other than English at home (%)**						
Yes	30.4	34.3	21.6	30.0	31.8	p=0.11
**Household devices**						
Smartphone only	16.4	18.9	12.5	18.8	6.4	p=0.23
Some other device or combo	82.5	79.5	87.5	79.7	93.6	
No device	1.1	1.6	0.0	1.4	0.0	
**Household internet service (%)**						
Cellular only	18.6	19.2	11.3	26.0	18.2	p=0.04
Another connection or combo	78.5	77.1	88.7	69.0	81.5	
No internet	2.1	2.1	0.0	5.1	0.0	
Skipped	0.8	1.6	0.0	0.0	0.3	
**Internet Availability and Reliability (%)**						
Often/always worried about availability or reliability	21.0	24.5	12.0	22.1	21.6	p=0.14
Not often/always worried	78.1	73.8	88.0	77.8	78.1	

© 2025 Hanmer J et al. *JAMA Network Open*.

Skipped	0.9	1.7	0.0	0.0	0.3	
**Digital Health Literacy Scale score (%)**						
Scale=12	51.7	48.9	62.8	43.6	57.7	p=0.02
9<=Scale<=11	24.5	22.2	24.9	27.0	32.1	
Scale<9	22.2	26.5	11.5	28.6	9.9	
Skipped	1.6	2.4	0.7	0.9	0.3	

© 2025 Hanmer J et al. *JAMA Network Open*.

Supplemental eTable 2. Prior acute care utilization in the overall sample and by latent class, with sample weights.

	**Whole Sample**	**Urgency focused**	**Continuity Focused**	**Cost Focused**	**In-person Visit Focused**	**Statistical testing** (Pearson chi2 statistics with Rao&Scott correction for survey design)
**Weighted respondents**	944	475.9	208.7	181.9	77.4	
**Ever used in-person PCP for sick child (%)**						
Yes	86.4	82.0	92.8	86.8	95.8	p=0.02
**Ever used PCP telemedicine for sick child (%)**						
Yes	29.1	30.3	33.0	26.4	17.5	p=0.27
**Ever used non-PCP telemedicine for sick child (%)**						
Yes	9.8	10.8	9.6	9.4	5.3	p=0.70
**Ever used UC/ED/Hospital for sick child (%)**						
Yes	53.9	47.1	63.0	54.0	70.4	p=0.009
**Ever used other source of care for sick child (%)**						
Yes	0.5	0.4	0.7	0.2	1.0	p=0.61
**None of these sources of care for sick child (%)**						
Yes	3.7	3.5	1.8	8.1	0.0	p=0.03

© 2025 Hanmer J et al. *JAMA Network Open*.
