# Supplementary material for: Parent Preferences for Acute Respiratory Tract Infection Care
Source: JAMA Netw Open. 2025 Aug 8;8(8):e2525904. doi: 10.1001/jamanetworkopen.2025.25904 (PMC12334952; doi:10.1001/jamanetworkopen.2025.25904)
Supplement: Supplement 2. — Data Sharing Statement [file jamanetwopen-e2525904-s002.pdf]

Data Sharing Statement

Hanmer. Parent Preferences for Acute Respiratory Tract Infection Care. *JAMA Netw Open*. Published August 08, 2025. doi:10.1001/jamanetworkopen.2025.25904

Data
Data available: Yes
Data types: Deidentified participant data, Data dictionary
How to access data: hanmerjz@upmc.edu
When available: With publication

Supporting Documents
Document types: None

Additional Information
Who can access the data: Any reasonable request
Types of analyses: Any purpose
Mechanisms of data availability: without investigator support
